# Toward a unified biological hypothesis for the BDNF Val66Met-associated memory deficits in humans: a model of impaired dendritic mRNA trafficking

**DOI:** 10.3389/fnins.2013.00188

**Published:** 2013-10-30

**Authors:** Gabriele Baj, Davide Carlino, Lucia Gardossi, Enrico Tongiorgi

**Affiliations:** ^1^Department of Life Sciences, Brain Centre for Neurosciences, University of TriesteTrieste, Italy; ^2^Brain Centre for Neurosciences, University of TriesteTrieste, Italy; ^3^Department of Medical, Surgical, and Health Sciences, Psychiatric Clinic, University of TriesteTrieste, Italy; ^4^Dipartimento di Scienze Chimiche e Farmaceutiche, University of TriesteTrieste, Italy

**Keywords:** neurotrophins, BDNF, memory deficits, post-traumatic stress disorder, hippocampus atrophy, dendritic mRNA trafficking, regulated protein secretion

## Abstract

Brain-derived neurotrophic factor (BDNF) represents promotesa key molecule for the survival and differentiation of specific populations of neurons in the central nervous system. BDNF also regulates plasticity-related processes underlying memory and learning. A common single nucleotide polymorphism (SNP) rs6265 has been identified on the coding sequence of human *BDNF* located at 11p13. The SNP rs6265 is a single base mutation with an adenine instead of a guanine at position 196 (G196A), resulting in the amino acid substitution Val66Met. This polymorphism only exists in humans and has been associated with a plethora of effects ranging from molecular, cellular and brain structural modifications in association with deficits in social and cognitive functions. To date, the literature on Val66Met polymorphism describes a complex and often conflicting pattern of effects. In this review, we attempt to provide a unifying model of the Val66Met effects. We discuss the clinical evidence of the association between Val66Met and memory deficits, as well as the molecular mechanisms involved including the reduced transport of BDNF mRNA to the dendrites as well as the reduced processing and secretion of BDNF protein through the regulated secretory pathway.

## BDNF and its Val66Met polymorphism

The development and plasticity of the nervous system are deeply influenced by a family of neurotrophic factors called neurotrophins. In mammals, the neurotrophin family consists of four related proteins: Nerve growth factor (NGF; Levi-Montalcini and Booker, 1960), Brain-derived neurotrophic factor (BDNF; Barde et al., 1982), neurotrophin-3 (NT3; Hohn et al., 1990) and neurotrophin-4 (NT4; Berkemeier et al., 1991). Since its discovery three decades ago, BDNF has been firmly implicated in the development of the nervous system in vertebrates and in the support of survival and differentiation of specific populations of neurons in the central nervous system (Bibel and Barde, 2000; Binder and Scharfman, 2004; Chao et al., 2006). More recently, BDNF has also emerged in several functions in the adult brain in which it is involved in the main plasticity-related processes including memory and learning (Tyler et al., 2002; Yamada et al., 2002).

A common single nucleotide polymorphism (SNP) rs6265 has been identified on the coding sequence of human *BDNF* located at 11p13 (Hall et al., 2003). This single base mutation, presenting an adenine instead of a guanine at position 196 (G196A), results in the amino acid substitution Val66Met (Hall et al., 2003). The polymorphism Val66Met only exists in humans and has been associated with a plethora of effects ranging from detrimental molecular, cellular and brain structural modifications associated with social and cognitive dysfunction (Dincheva et al., 2012). While the literature on this polymorphism is rapidly increasing, there is little consensus on the pattern of results. In this review, we discuss how this single nucleotide variant affects molecular mechanisms of memory formation and maintenance and summarize clinical evidence on the association between Val66Met SNP and memory deficits. In conclusion, we hypothesize a biological mechanism underlying the memory deficits associated with the Val66Met polymorphism of BDNF.

## Clinical aspects of Val66Met

The first association between the BDNF Val66Met SNP and a clinical phenotype was reported in schizophrenic patients, their relatives and healthy controls (Egan et al., 2003). This study showed a specific effect on cognitive functions where the Met BDNF allele reduced the delayed recall of episodic memory in all three groups but had no influence on other cognitive domains or *intelligence quotient* (IQ). Both Egan et al. (2003) and Hariri et al. (2003) demonstrated that Met BDNF carriers displayed reduced hippocampal engagement during encoding and retrieval of a spatial task with respect to Val/Val homozygotes. Two independent MRI investigations (Pezawas et al., 2004; Szeszko et al., 2005) extended the findings of Egan and Hariri (Egan et al., 2003; Hariri et al., 2003) by demonstrating in affectively ill individuals that Val/Met heterozygotes displayed lower hippocampal volumes than their Val/Val counterparts. In addition, there was volume reduction of the gray matter in the dorsolateral prefrontal cortex (DLPFC), which is implicated in learning and memory processes involving also the hippocampus (Pezawas et al., 2004; Hwang et al., 2006; Benjamin et al., 2010). Further, in a community of elderly Caucasian individuals, BDNF Val/Val homozygotes performed significantly better compared to both Val/Met heterozygous and Met/Met homozygous individuals on a delayed recall task and an alphabet-coding task (ACT), a measure of processing speed. Another study also noted a decrease in hippocampal volume in Met BDNF allele carries, although it did not reach statistical significance (Goldberg et al., 2008) while other authors confirmed the hippocampal volume reduction (Benjamin et al., 2010; Teh et al., 2012; Tost et al., 2013).

In a fascinating study, Schofield et al. (2009) found a significant increase in hippocampal activity in Met BDNF carriers compared to BDNF Val/Val homozygotes during the auditory oddball task. The auditory oddball task has been asserted to measure auditory attention and auditory capacity. It is a task in which the subject must detect a relevant (“oddball”) stimulus, which is presented infrequently and randomly within a train of task-irrelevant stimuli. Schofield and colleagues argued that Met BDNF carriers might require greater hippocampal activation than their BDNF Val/Val counterparts to process auditory stimuli presented during the task, inadvertently depleting resources for prefrontal processing of stimuli. Similar circuit dysregulation has been observed previously. A protective role for the Met allele has also been proposed for enhanced verbal reasoning in the elderly (Harris et al., 2006), systemic lupus erythematosus (Oroszi et al., 2006), multiple sclerosis patients (Zivadinov et al., 2007; Cerasa et al., 2010), and Parkinson's disease (Foltynie et al., 2005), although others have challenged these findings (Liguori et al., 2007; Miyajima et al., 2008).

Additional studies found either a gender-specific association (Echeverria et al., 2005) or no association in schizophrenic and bipolar patients (Strauss et al., 2004; Tramontina et al., 2009) as well as in other clinical conditions characterized by cognitive impairment such as multiple sclerosis (Cerasa et al., 2010), HIV-associated neurocognitive disorders (Levine et al., 2012) and Parkinson's disease (Guerini et al., 2009). The disagreement between results could be due to a number of differences in study design (Hong et al., 2011). First, these studies are typically characterized by relatively small sample size. Second, there is a noticeable variability in the phenotypes analyzed in these researches. Specifically, it is striking how many different tasks have been used in the field to assess arguably similar (or the same) cognitive functions. In animal studies, BDNF is studied especially in the hippocampus and therefore, the impact of the Val66Met polymorphism could have been more evident in studies focused on hippocampus-specific tasks. More orchestrated approaches to behavioral phenotyping for cognition in the design of genetic and allelic association studies on BDNF are highly needed and are likely to be fruitful. Otherwise, using the mere Mini Mental State Examination (MMSE), the effects of BDNF Val66Met polymorphism on cognition may not emerge (Zivadinov et al., 2007; Forlenza et al., 2010).

More recently, Mandelman and Grigorenko (2012) conducted a meta-analysis examining the relationship between BDNF Val66Met and cognition, but did not find significant associations between the Val66Met polymorphism and any of the phenotypes that were included. The obvious additional candidates for sources of such between-study heterogeneity are demographic characteristics such as gender, age and phase of illness, ethnicity, physical exercise, cardio-vascular health status and diagnosis (Verhagen et al., 2010; Lu et al., 2012; Martinho et al., 2012; Nagata et al., 2012; Smith et al., 2012). Furthermore, it is possible that the genetic variant may be associated with some intermediate phenotypes, which, in turn, could be related to some but not all of the cognitive phenotypes examined. Given the multitude of BDNF protein isoforms and the diversity of its transcripts in different brain areas, it is conceivable that cognitive phenotypes should be grouped not by their behavioral similarities, but by similarities in the brain activation pathways that underlie these phenotypes.

## Biological aspects of the Val66Met mutation

It is important to highlight that the vast majority of the results reported in the literature and summarized here, were obtained by investigations on homozygous BDNF Met/Met (Met BDNF) mice while the double mutated Met/Met allele is quite rare in humans. Indeed, data from human population are collected mostly from individuals with BDNF Val/Met (single allele mutation) or BDNF Val/Val (Mandelman and Grigorenko, 2012). The biological effects of the SNP rs6265 in the BDNF coding sequence have been studied using several different model systems, starting from cell culture, to animal models or in correlation with diseases affecting the human population. In their initial work, using an *in vitro* neuronal culture system, Egan and coworkers (Egan et al., 2003) demonstrated that Met BDNF in fusion with GFP and transfected in neurons is produced at levels similar to the control Val BDNF-GFP. The knock-in animal model for the Met BDNF allele created by Chen et al. (2006; Dincheva et al., 2012) confirmed that the total BDNF production is not affected by the polymorphism. A recent detailed analysis of BDNF protein in specific brain areas of BDNF Val and Met knock-in mice showed slight but significant reduction of BDNF in the hippocampus (HPC) and in prefrontal (PFC) cortex while no variations were found at the level of amygdala and striatum (Bath et al., 2012; Yu et al., 2012).

Chen and coworkers were first to describe the functional importance of the Met BDNF allele in the brain. They reported alterations in hippocampal anatomy of the Met BDNF knock-in model. The total hippocampal volume of Met BDNF mice was found to be reduced and dentate gyrus (DG) neurons were measured as significantly smaller in total volume and dendritic complexity while the soma size was not affected (Chen et al., 2008). Similar neuronal volumetric reduction was reported also on ventromedial prefrontal cortex cells, while neurons from the striatum were not affected (Yu et al., 2009). The same mouse model was used to establish the effect of Met polymorphism in prefrontal cortex (PFC) where a specific atrophy of apical dendrites of layer 5 pyramidal cells was found (Liu et al., 2012). Furthermore, the induction of synaptogenesis in PFC by ketamine administration on brain slices was almost abolished indicating impaired synaptic formation/maturation. Met BDNF mice presented alterations in the generation of LTP at the CA3-CA1 synapse. Ninan and colleagues were able to dissect out the mechanism of this deficit revealing that NMDA receptor-dependent LTP was specifically affected while the non-NMDA receptor neurotransmission (i.e., mGluR) was normal (Ninan et al., 2010; Pattwell et al., 2012). Yet, it remains unclear if these BDNF effects on the NMDA receptor are either due to a deficit in the basal regulation of the NMDA receptor trafficking and expression or to an altered acute activity-dependent release of BDNF, in Met BDNF mice. Additional evidence of deficits in neuronal activation was observed in ventromedial prefrontal cortex of Met BDNF mice following fear extinction tests: the Met substitution results in reduced cellular activation (–50%), visualized by cFOS positivitity (Yu et al., 2009).

## Effects of Val66Met on BDNF secretion and trafficking

Apart from of the evidence of a normal (total brain) or reduced (HPC and PFC) production of BDNF in presence of the Val66Met mutation, several studies reported a differential subcellular distribution of BDNF upon Met substitution. BDNF was shown to regulate synaptic strength in a site-restricted manner and therefore, its subcellular distribution is very important for the functional role of this neurotrophin (Steward and Schuman, 2001; Horch and Katz, 2002; Lu, 2003; Alonso et al., 2004; Horch, 2004). Given the morphological structure of neurons, highly polarized cells with a relatively small soma and long dendritic/axonal processes, the BDNF protein must overcome several challenges to exert its functions correctly. In particular, just like many other synaptic proteins, BDNF is synthesized in response to synaptic stimuli and needs to be delivered at synapses located in specific subcellular districts where it can modify their structure and function (Tongiorgi, 2008; Edelmann et al., 2013). Jiang and Schuman reviewed the three different models that explain how a protein can be selectively delivered at activated synapses (Jiang and Schuman, 2002). In one model, the protein is synthesized in the soma and becomes tagged for a specific group of synapses, where it is subsequently transported. The second model describes the protein as being transported to dendrites without a specific target but is then captured by “tagged” synapses which have been appropriately stimulated. In the third model, which is in contrast with the previous two, it is the mRNA that is transported to dendrites and where it is locally translated by the protein synthesis machinery localized in proximity to synapses. Most researchers in the field agree that these three mechanisms might coexist within the same cell and may even regulate the very same protein.

BDNF represents one clear example of coexistence of multiple mechanisms for dendritic trafficking. The mRNAs encoding for BDNF were found to be transported and actively accumulate along dendrites following stimulation of electrical activity (Tongiorgi et al., 1997, 2004; Jakawich et al., 2010; Baj et al., 2013). BDNF was also shown to be transported to dendrites in large, dense-core secretory vesicles (Edelmann et al., 2013). Accordingly, recent studies have investigated the possible impact of the Met polymorphism on these BDNF sorting mechanisms.

In neurons, Met BDNF seems to be preferentially produced and accumulated within the somatic district and only partially transported in the proximal area of the primary dendrites whereas Val BDNF is produced and transported also in secondary and tertiary dendrites (Egan et al., 2003; Chen et al., 2004, 2006). The abnormal distribution of Met BDNF in cell body and dendrites of hippocampal neurons appears to be accompanied by a significant reduction in the secretion of this neurotrophin. In particular, secretion of BDNF induced in response to electrical stimuli is compromised (30% decrease) while the constitutive secretion of BDNF is not affected (Egan et al., 2003; Chen et al., 2004, 2006). BDNF sorting into the regulated secretory pathway appears to be mediated by two mechanisms. The first, involves interaction of the sorting receptor carboxypeptidase-E (CPE) with a tetrad of aminoacids (I16, E18, I105, D106) in the mature BDNF region forming a specific recognition motif, which is not affected by the Val66Met mutation (Lou et al., 2005). The second sorting mechanism, regards the interaction of sortilin with the pro-BDNF region comprised between aminoacid 44 and 102, wherein the Val66Met occurs (Chen et al., 2005). The current view is that substitution of the Val66 with a Met causes less efficient interaction with sortilin and therefore Met BDNF proteins show decreased targeting to the regulated secretory pathway (Dincheva et al., 2012). Further evidence in support of this hypothesis was provided by experiments in which Met BDNF was able to impair the trafficking of BDNF Val following formation of BDNF Val and Met BDNF heterodimers (Chen et al., 2004).

The combinatorial effect of normal BDNF production but decreased dendritic localization and secretion is correlated with the reduction of BDNF in SecII-positive secretory vesicles and Synaptophysin-positive synapses (Egan et al., 2003). The different biochemical pathways involved in the production and sorting of BDNF was also specified by Del Toro et al. (2006), who not only confirmed the reduction of Met BDNF in SecII vesicles but also found an accumulation of Met BDNF in Giantin-positive Golgi vesicle. Moreover, they found no visible differences between Val BDNF and Met BDNF in calnexin-positive vesicles while the total number of vesicles containing BDNF was strongly reduced by about 30% (Del Toro et al., 2006). It is important to note that the polymorphism at position 66 in the BDNF pro-domain does not affect the cleavage of the pro-BDNF form and the generation of the mature form of BDNF (Chen et al., 2004).

Transport dynamics of Met BDNF and Val BDNF through the secretory pathway are comparable. Indeed, the calculation of the mean translocation velocity of secretory vesicles in dendrites showed no differences between the two alleles (Del Toro et al., 2006), suggesting that the transported BDNF protein may probably account for the constitutive secretion of this neurotrophin which is also not affected by the Val66Met polymorphism. However, the physiological role of BDNF is only partially supported by this basal BDNF secretion. In fact, Bath et al. (2008) showed that TrkB activation in neurogenic regions, revealed by the intensity of pTrkB staining on total TrkB (in SVZ and OB), was reduced by 30% in Met BDNF brains. These data point out that activity-dependent BDNF secretion is required for a physiological activation of TrkB to sustain neurogenesis and neuroblast survival. Accordingly, a recent characterization of the neurogenesis at the level of the DG in Met BDNF mice, calculated as density of BrdU positive cells, found a considerable reduction (ca. –25%) of proliferating neural progenitors (Bath et al., 2012).

## Effects of BDNF Val66Met: an mRNA perspective

**Figure 1:**
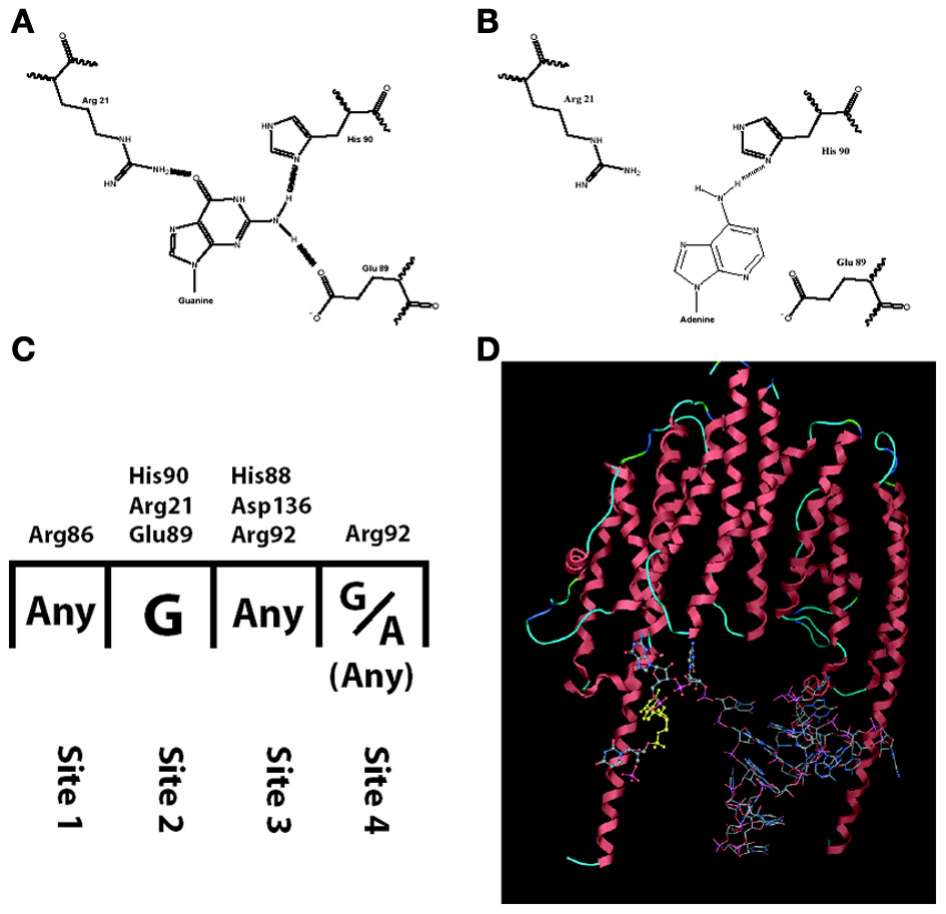
**Interaction between translin and human BDNF mRNA is hampered by the mutation G196A. (A)** Translin amino acids His-90, Arg-21, Glu-89 form three hydrogen bonds with G196 (normal human BDNF) but only one with A196 (mutated BDNF, in **B**). **(C)** The nucleotide: amino acid selectivity for each of the four binding sites on translin. **(D)** Three-dimensional modeling of translin dimer binding to human BDNF mRNA.

Among the studies that report a reduced amount of dendritic BDNF protein in presence of the Val66Met polymorphism, one study compared the trafficking of BDNF mRNA with the wild type sequence (Val allele: G196) or with the mutation (Met allele: A196). The results revealed significantly reduced dendritic trafficking of BDNF mRNA bearing the A196 mutation (Chiaruttini et al., 2009). This effect was found to be due to a reduced affinity of the RNA-binding protein Translin for the mutated allele. The RNA-binding domain of Translin contains a pocket made by three aminoacids (His90, Arg21, Glu89), which specifically recognizes a guanosine by forming three hydrogen bonds (Figures 1A,C). The transition from Guanine to Adenine at position 196 reduces the number of possible hydrogen bonds to just one, with a dramatic reduction in binding stability of Translin to the mutated BDNF mRNA (Figure 1B). Since the minimal tertiary configuration with which Translin can bind to an mRNA is a dimer, the authors hypothesized that there could be a second Guanine on BDNF mRNA that binds to the other Translin participating to the dimer (Figure 1D). Indeed, a second Guanine was identified at position 177 and when mutated to an Adenine, it abolished the Translin-mediated trafficking in dendrites exactly like the Guanine at position 196 (Chiaruttini et al., 2009). This mutation of Guanine at position 179 has no effect. Translin is involved in the trafficking of other transcripts associated with synaptic functions such as the mRNA encoding for CamK-II and the small non-coding RNA BC-1, implicated in regulating protein translation in dendrites (Li et al., 2008; Jaendling and McFarlane, 2010). In addition, Translin can associate with a similar protein called TRAX to form a complex known as C3PO, which is highly conserved from fungi to humans (Tian et al., 2011; Ye et al., 2011). This complex is a component of the RISC complex and is required for the mechanisms of RNA interference, which regulates mRNA stability and translation at synapses. Remarkably, Translin KO-mice have several behavioral abnormalities (Li et al., 2008; Jaendling and McFarlane, 2010). Thus, abnormal interactions of an mRNA with Translin may have profound effects on its trafficking, stability and translation (Wu et al., 2011).

A recent study in humans found a strong correlation, defined by linkage disequilibrium, among rs6265 and two other SNPs in the 3′UTR of BDNF (rs11030100 and rs11030099). All these SNPs transform BDNF mRNA into a target for the miR-26s family providing a supplementary component involved in regulation of mRNA stability and translation (Caputo et al., 2011). These pieces of evidence are consistent with findings in BDNF Met/Met mice and suggest that the reduced availability of BDNF mRNA in dendrites of Met BDNF neurons leads to a deficiency of local protein translation and may contribute to deficits in activity-dependent release of Met BDNF from post-synaptic terminals.

## Toward a unified biological hypothesis for the Val66Met deficit

At the current state of research, a number of studies support the view that the BDNF Val66Met polymorphism, with one or two copies of the Met BDNF allele, can lead to altered performance of learning and memory functions, especially with impairments in hippocampal and cortical processes, suggestive of reduced neuroplasticity. To better understand how a small change in the BDNF sequence may determine so many effects, we believe that it is worth trying to assemble the large body of data available in the literature into a unified model at the cellular, anatomical, and behavioral level.

From a cellular standpoint, converging studies suggest that neuroplasticity deficits can be accounted mainly by impairment of activity-dependent translation and release of the Met BDNF from post-synaptic sites. We now describe how this deficit can result from a combination of reduced transport of Met BDNF mRNA in dendrites and an altered processing of the mutated protein through the regulated secretory pathway.

**Figure 2:**
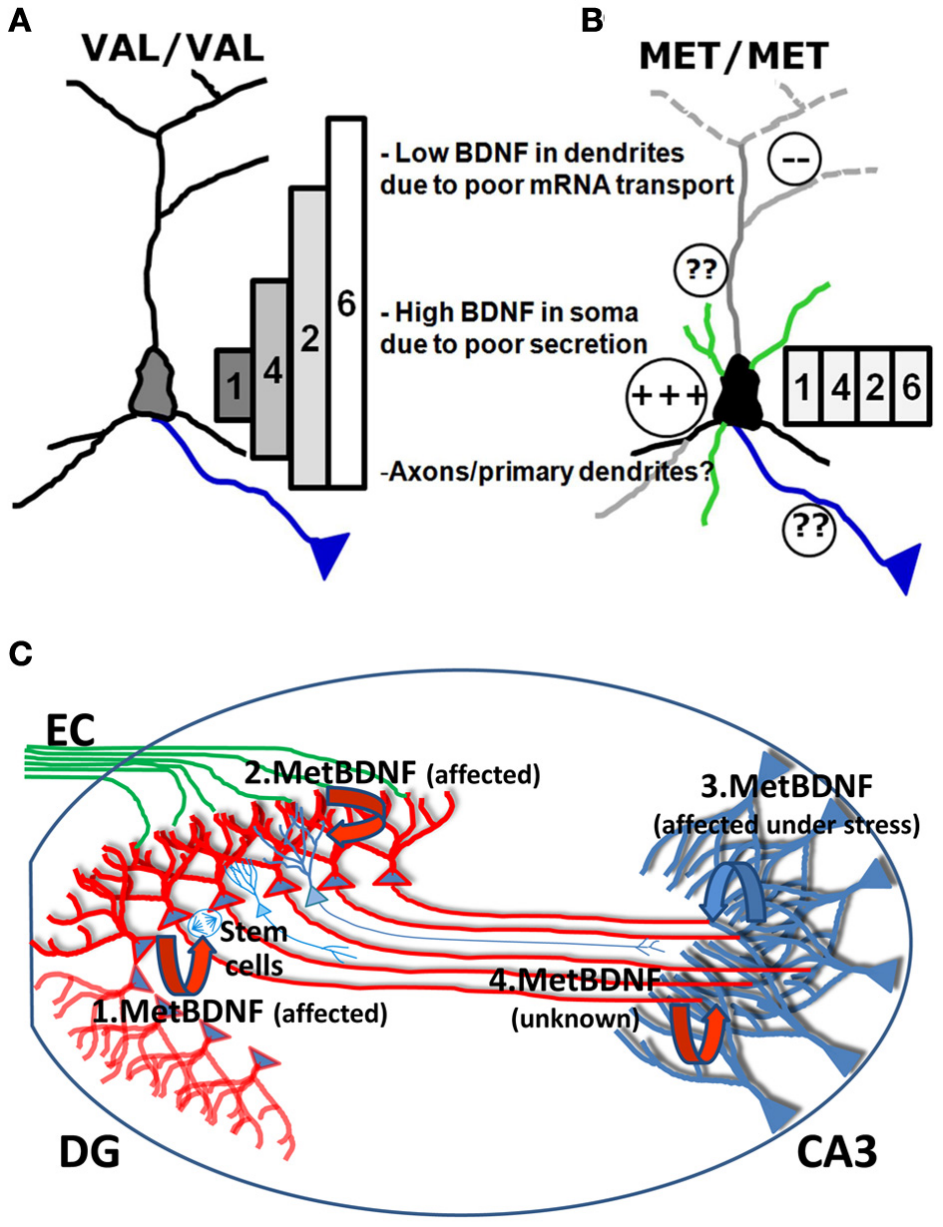
**Model of the biological effects of Val and Met BDNF alleles in the hippocampus. (A)** In Val/Val neurons, the four most abundant brain BDNF transcripts, encoding exon 1, 2, 4, or 6, are differentially localized in dendrites, forming a gradient with exon 1 being restricted to the soma and exons 2, 4, and 6 in dendrites at increasing distances according to the gradient 4<2<6. Exons 1 and 4 can affect only the morphology of proximal dendrites while exon 2 and 6 are able to shape the distal dendrites. **(B)** In Met/Met neurons, transport of BDNF mRNA in distal dendritic districts is impaired and all BDNF mRNA isoforms accumulate in the cell soma. Met BDNF protein increases in the soma, also due to poor secretion. It is unclear if axonal transport and secretion can be affected by the Met mutation. **(C)** BDNF released from DG granule cells is shown in red, while BDNF released from CA3 neurons is shown in blue. (1.BDNF) Secretion of Met BDNF from the soma of DG granule cells is altered and provides insufficient local trophic support for survival and differentiation of DG subgranular neural stem cells. (2.BDNF) Release of Met BDNF from dendrites of DG neurons is affected and cannot provide sufficient autocrine support to dendrites which show reduced arborization. (3.BDNF) Local production and release of Met BDNF from apical dendrites of CA3 neurons is reduced and cannot provide the target-derived trophic support to promote innervation of CA3 from newly formed mossy fibers and autocrine support to CA3 dendrites. (4.BDNF) It is unknown if anterogradely transported Met BDNF in mossy fibers can support dendrites from CA3 neurons and if anterograde transport of BDNF is affected by the Met mutation.

The deficit in mRNA trafficking found in BDNF bearing the Met allele (G196A at mRNA level), can be put into the broader perspective of the so called “spatial code model of BDNF transcripts” (Chiaruttini et al., 2008; Tongiorgi, 2008). BDNF is encoded by multiple mRNAs generated by alternative splicing. Eleven non-coding 5′UTR exons are alternatively spliced to a common downstream exon containing the coding region and a 3′UTR with two polyadenylation signals (An et al., 2008) which produce 11 different transcripts in rodents and 14 in humans, each with two 3′ tails (Aid et al., 2007; Pruunsild et al., 2007). Following a series of studies demonstrating the differential subcellular distribution of the different BDNF transcripts (Pattabiraman et al., 2005; Chiaruttini et al., 2008), a hypothesis was put forward that the multiple transcripts encoding the very same protein are used to generate a spatial code for expressing BDNF at restricted subcellular locations, leading to localized effects (Tongiorgi, 2008). This model was recently completed by real-time PCR analysis of all rodent transcripts *in vivo* (Baj et al., 2013). In untreated rats, various BDNF transcripts were detected in dendritic fields of the hippocampus (exons 6 and 7 in CA1; exons 1, 6, and 9a in CA3; and exons 5, 6, 7, and 8 in DG). However, due to the very low levels of most of these transcripts, the exon 6 was found to be the main transcript present in dendrites at resting conditions. Strong neuronal activation by pilocarpine-induced *status epilepticus* caused an increase in all hippocampal dendritic laminae of BDNF transcripts encoding exons 2, 4, and 6 and also of BDNF exons 3 and 9a in DG molecular layer, whereas the other transcripts were restricted to the soma (Baj et al., 2013). Importantly, overexpression or silencing of the four most abundant brain BDNF transcripts, encoding exon 1, 2, 4, or 6, led to differential effects on dendritic morphology *in vitro*, with exons 1 and 4 affecting only the proximal dendritic domains and exon 2 and 6 being able to shape the distal dendritic district (Baj et al., 2011). This differential effect was demonstrated to be due to localized expression of BDNF and activation of TrkB receptor in the same subcellular domains where the specific mRNAs are localized (Baj et al., 2011). The conversion of the G196 to A196 impairs the transport of BDNF mRNA into dendritic domains (Chiaruttini et al., 2009), and therefore, the mutated BDNF mRNAs can only be translated in the soma. Since the change G196A is located within the coding region, it is present in all BDNF mRNA variants but its effects on mRNA trafficking are only evident for those BDNF transcripts that are actively transported to dendrites (such as exon 2 or 6). However, activity-dependent secretion is also impaired, causing entrapment of the Met BDNF protein within the Golgi apparatus and the observed increase in the soma and depletion from distal dendrites (Egan et al., 2003; Chen et al., 2004) (Figure 2). In this model, the converging misplacement of BDNF mRNA and protein can affect the ability of BDNF to maintain dendritic branching in the periphery of the neuron and support plasticity at synapses in distal dendrites which represent, notably, the large majority of glutamatergic excitatory synapses (Figures 2 A,B).

Anatomically and physiologically, the available findings are consistent with morphological and functional hippocampal atrophy and subsequent cortico-cortical disconnection syndrome, which involves the disruption of neural networks between the anterior and posterior cerebral areas (Delbeuck et al., 2003; Nagata et al., 2010). Met carriers, rather than subjects with the Val/Val phenotype, may benefit from a protective role on executive function through hippocampus cortical atrophy or other subcortical tract changes, as reported in previous studies on elderly people. This observation may in part, explain the reason why this polymorphism despite its deleterious effects remains highly diffused in the world population. Interestingly, as pointed out by Li et al. (2010), the negative effects of the BDNF polymorphism on episodic memory are most likely observed when associative and executive demands are high. These observations are in line with the hypothesis that the magnitude of genetic effects on cognition is greater when brain resources are reduced, as with old age. Accordingly, the logical consequence of the strong effects of Met BDNF allele on mental performance are pronounced in the elderly, with poor or no impact on reproduction and therefore, transmission of the allele to the following generations can be ensured thus contributing to the maintenance of this mutation through the population. In addition, carriers of the Met allele are more likely to develop post-traumatic stress disorder after traumatic experiences.

In Figure 2C we summarize how deficits in Met BDNF secretion and transport, observed at the cellular level, may affect the hippocampal neuronal circuit. According to this model, since various studies reported that survival and differentiation of DG subgranular neural stem cells are reduced, secretion of Met BDNF from the soma of DG granule cells is altered and provides insufficient local trophic support (Figure 2). In addition, since DG dendrites were shown to have reduced arborization, the model predicts that release of Met BDNF from dendrites of DG neurons is affected and cannot provide sufficient autocrine support. Following stressful situations, Met/Met individuals may undergo more easily to atrophy of apical dendrites of CA3 neurons. According to our model, the local production and release of Met BDNF from apical dendrites of CA3 neurons is reduced and thus cannot provide target-derived trophic support to promote the innervation of CA3 from newly formed mossy fibers and autocrine support to CA3 dendrites. It is currently unclear if Met BDNF present in mossy fibers can support dendrites from CA3 neurons and if anterograde transport of BDNF is affected by the Met mutation. One plausible hypothesis is that BDNF released from mossy fiber axons may stimulate dendritic targeting of BDNF mRNA in CA3 apical dendrites, thus contributing to a local autocrine loop which supports the maintenance of apical dendritic arborization.

In conclusion, the view proposed here does not claim to be the ultimate interpretation of the available data but intends to be a stimulus for the scientific community to develop a consensus model on the biological mechanisms of one of the most fascinating human mutations affecting cellular functions, brain morphology, and cognition.

### Conflict of interest statement

The authors declare that the research was conducted in the absence of any commercial or financial relationships that could be construed as a potential conflict of interest.
